# Somatic Mutation Trajectories Define Prognostically Distinct Subtypes and Shape the Tumor Microenvironment in Gastric Cancer

**DOI:** 10.3390/genes17050536

**Published:** 2026-04-30

**Authors:** Yikang Shen, Huaxin Pang, Haiyu Liu, Pengzhen Ma, Mingrui Liu, Yaning Li, Qihao Wang, Xiaoxia Xie, Xiaoping Zhang, Yufeng Zhao

**Affiliations:** Data Center of Traditional Chinese Medicine, China Academy of Chinese Medical Sciences, Beijing 100700, China; shenyikangcm@163.com (Y.S.); 20112005@bjtu.edu.cn (H.P.); haiyu_liu2001@163.com (H.L.); mpz1993@126.com (P.M.); 15345986601@163.com (M.L.); xiao_ping_zhang@139.com (X.Z.)

**Keywords:** somatic mutation trajectories, gastric cancer, tumor evolution, tumor microenvironment, computational genomics

## Abstract

**Objective:** Gastric cancer (GC) is characterized by molecular heterogeneity, yet current classifications are largely based on cross-sectional molecular profiles and do not account for the temporal order of mutation accumulation. This study aimed to reconstruct somatic mutation trajectories to identify prognostically distinct subtypes and to examine transcriptomic and microenvironmental features associated with these inferred trajectories. **Methods:** We applied the Subtype and Stage Inference (SuStaIn) algorithm to TCGA-STAD somatic mutation data to infer the temporal sequence of mutation accumulation. Stage-correlated gene expression analysis was performed to identify genes whose expression levels changed with evolutionary stage. The tumor microenvironment (TME) was characterized using EcoTyper and single-cell RNA sequencing deconvolution, while drug sensitivity was estimated through transcriptome-based IC_50_ prediction. The clinical relevance of the inferred trajectories was further evaluated in three independent external transcriptomic cohorts. **Results:** We identified two distinct evolutionary trajectories: the Accelerated Path (AP, 65%) and the Gradual Path (GP, 35%). In the AP, *TP53* mutations were positioned at an earlier evolutionary stage (Stage 3) compared to the GP (Stage 8). AP patients had significantly worse overall survival (Hazard Ratio = 1.437, *p* = 0.044, adjusted for clinical stage and molecular subtypes). The AP was associated with stage-correlated downregulation of the sodium channel gene *SCN4A* (ρ = −0.36, *p* < 0.001) and an increase in a squamous-associated gene expression score, while the GP showed stage-correlated expression changes in the mitochondrial gene *SDHD* (ρ = −0.35, *p* < 0.001). The AP was further characterized by higher inferred abundance of extracellular matrix CAFs (eCAFs) and lower inferred immune cell scores, whereas the GP was associated with higher inferred signatures of activated B cells and effector memory T cells. Computational drug sensitivity modeling predicted a negative correlation between AP stage and IC_50_ values for 5-Fluorouracil and Docetaxel. **Conclusions:** Two distinct mutational ordering patterns identified by SuStaIn are associated with divergent transcriptomic features, TME compositions, and clinical outcomes in gastric cancer. The AP subtype is characterized by early *TP53* mutations, *SCN4A* downregulation, and a stromal-enriched microenvironment, while the GP subtype is associated with later *TP53* mutations, *SDHD*-correlated expression, and higher inferred immune cell scores. The reproducibility of these associations was confirmed in independent cohorts. The computational drug sensitivity predictions and the proposed mechanistic links between gene expression patterns and clinical outcomes should be viewed as hypothesis-generating findings that require prospective and functional validation.

## 1. Introduction

Gastric cancer (GC) remains one of the most lethal malignancies worldwide [1], characterized by high inter-tumor heterogeneity and complex molecular landscapes [2,3,4,5,6,7,8,9,10]. While landmark classification systems like the Lauren or TCGA molecular subtypes have significantly advanced our understanding of the disease’s static features, they often fail to capture the temporal dimension of tumor progression [11,12]. Tumors with similar static molecular profiles frequently exhibit disparate clinical outcomes and therapeutic responses [13,14,15,16], suggesting that the chronological order and velocity of molecular events—rather than their mere presence—may dictate a tumor’s ultimate biological destiny.

In the paradigm of cancer evolution, somatic mutations do not accumulate randomly; they follow preferred trajectories that reflect the selective pressures of the tumor microenvironment (TME) [17,18,19]. However, reconstructing these temporal sequences in clinical cohorts is historically challenging due to the lack of longitudinal sampling [20,21,22,23]. Recent advances in machine learning, specifically the Subtype and Stage Inference (SuStaIn) algorithm, have made it possible to map the most probable sequences of mutation accumulation within “pseudo-time” stages [24,25,26]. This evolutionary perspective allows us to transition from “what” mutations are present to “when” they occur and “how” their timing shapes the tumor’s phenotypic identity.

In this study, we hypothesize that the temporal accumulation of somatic mutations in GC bifurcates into distinct evolutionary paths, each associated with a unique program of transcriptomic and microenvironmental remodeling. We identified two distinct trajectories: the AP, where *TP53* mutations were inferred at early stages, and the GP, where *TP53* mutations were assigned to later stages in the model.

Our findings reveal a potential neural-squamous transition in the AP trajectory, characterized by the bioelectric alterations of the sodium channel *SCN4A* and a significant increase in squamous identity scores, which together correlate with aggressive invasion. Conversely, the GP trajectory maintains a metabolically distinctive identity marked by the relative preservation of mitochondrial integrity, represented by *SDHD*. Furthermore, we demonstrate that these evolutionary routes are associated with diametrically opposite TME landscapes—a fibrotic, CAF-rich “cold” environment in the AP and an immunologically active “hot” environment in the GP.

**Figure 1:**
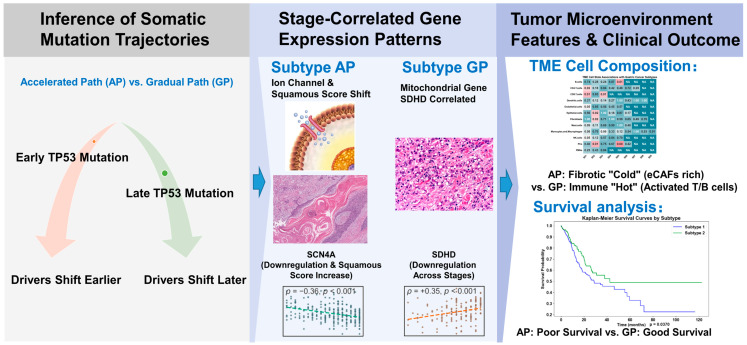
Workflow of the study design.

Finally, we explore the clinical implications of these paths, identifying computational evidence of an “evolutionary vulnerability” in the AP that predicts a potential sensitivity to traditional chemotherapy during specific evolutionary stages. By defining these trajectories, we offer a dynamic roadmap for integrating evolutionary history into the computational framework of precision medicine for gastric cancer. The overall workflow of the study design is illustrated in Figure 1.

## 2. Materials and Methods

### 2.1. Data Sources and Preprocessing

Data were retrieved from The Cancer Genome Atlas (TCGA) stomach adenocarcinoma cohort (TCGA-STAD), including somatic mutation data, RNA expression data, TNM staging, and survival data (http://xena.ucsc.edu/). After quality control, 400 patients with complete survival and TNM staging data were included in the analysis.

Somatic Mutation Data: Somatic mutations (single nucleotide polymorphisms [SNPs] and small insertions/deletions [INDELs]) were sourced from the Ensemble Somatic Variant (WXS) pipeline in TCGA. Preprocessing was performed to meet the requirements of the Subtype and Stage Inference (SuStaIn) algorithm [25], which assumes monotonic changes in input features due to the irreversible nature of somatic mutations. Genes with a mutation frequency greater than 150 across the dataset were selected, resulting in six genes: *TTN*, *MUC16*, *TP53*, *LRP1B*, *OBSCN*, and *ARID1A*. To address data sparsity for lower-frequency genes, a literature review was conducted to identify additional gastric cancer-associated genes and gene families [27,28,29,30]. Related genes were grouped into functional families based on shared pathways (e.g., *CDH1*, *CDH2*, and *CDH3* into the *CDH* family). This approach yielded 11 additional gene families: *SYNE*, *FAT*, *CSMD*, *CDH*, *PCDH*, *DNAH*, *NOTCH*, *ERBB*, *FGF*, *KMT*, and *BMP*. Detailed gene-to-family mappings are provided in Supplementary Table S1.

RNA expression data were obtained as STAR-Counts from the TCGA repository. Raw counts were used as input for downstream analyses. Normalization was performed within each method-specific pipeline. For differential expression analysis, DESeq2 internal normalization (median-of-ratios method) was applied. For correlation-based analyses (e.g., WGCNA), Variance Stabilizing Transformation (VST) was used.

Cross-cohort comparisons were performed using log2(TPM + 1) transformed data, and batch effects across datasets were assessed and minimized using z-score standardization.

To minimize the potential bias from passenger mutations associated with gene length (*TTN*, *MUC16*), we conducted a sensitivity analysis by re-running the SuStaIn algorithm after excluding these features. The results demonstrated that the evolutionary trajectories and subtype bifurcations remained highly consistent, ensuring the robustness of our classification (see Supplementary Table S4).

Clinical Data: TNM staging and survival data included tumor stage, lymph node involvement, metastasis status, overall survival time (in months), and survival status. These were used as provided after ensuring completeness for all 400 patients.

Missing data handling: Samples with incomplete survival or TNM staging information were excluded prior to analysis (*n* = 400 retained). For molecular data, genes with missing expression values in more than 20% of samples were removed; remaining missing values (if any) were not imputed, as RNA-seq count matrices from TCGA are generally complete after preprocessing.

Tumor purity: Tumor purity estimates were obtained from TCGA (ABSOLUTE/ESTIMATE where available). Sensitivity analyses were conducted by including tumor purity as a covariate in downstream differential expression models; results remained consistent, and thus primary analyses were reported without adjustment.

### 2.2. Subtype Classification Using SuStaIn Algorithm

The Subtype and Stage Inference (SuStaIn) algorithm [25,26] was used to identify gastric cancer subtypes based on somatic mutation progression sequences. The event-based model of SuStaIn was employed, designed for analyzing the sequential accumulation of somatic mutations. The input was a mutation matrix, with rows representing tumor samples and columns indicating the cumulative frequency of mutations in selected genes and gene families. SuStaIn was configured to assume monotonic mutation accumulation, generating subtypes characterized by distinct temporal mutation progression sequences.

The SuStaIn algorithm was employed based on the monotonic accumulation assumption, which aligns with the biological nature of cancer evolution. In cancer genomics, somatic mutations are considered irreversible genomic events; once a mutation occurs in a founding clone, it is passed down to all its progeny, representing a cumulative temporal process. Thus, the cross-sectional mutational burden per patient can be effectively utilized as a proxy for disease progression, allowing for the reconstruction of high-resolution evolutionary trajectories.

Feature selection (mutation genes and gene families) was performed exclusively on the TCGA-STAD cohort and frozen prior to all downstream validation analyses to prevent information leakage.

SuStaIn implementation details: The pySuStaIn framework was used with the event-based model. The number of subtypes was determined by comparing models with 1–5 subtypes using cross-validation likelihood, with the optimal model selected based on maximum likelihood and model stability. Model fitting was performed using Markov Chain Monte Carlo (MCMC) sampling with 100,000 iterations and 10,000 burn-in steps.

### 2.3. Differential Gene Expression and Pathway Enrichment

Differential Expression Analysis: RNA sequencing data from the TCGA-STAD cohort were analyzed using DESeq2 (DESeq2 Bioconductor) [31] to identify differentially expressed genes (DEGs) between subtypes defined by SuStaIn. RNA sequencing data were analyzed using DESeq2 (version ≥1.38). The following parameters were used: Size factor normalization: median-of-ratios; Dispersion estimation: parametric fit; Statistical test: Wald test; Design formula: ~Age + Sex + Subtype.

Pathway Enrichment Analysis: Gene set enrichment analysis (GSEA) [32] was performed using the GSEA software (version 4.3.2; Broad Institute, Cambridge, MA, USA) to identify enriched pathways. Input included DEGs identified by DESeq2, with pathways sourced from the Molecular Signatures Database (MSigDB) [33], including Gene Ontology (GO) [34], Kyoto Encyclopedia of Genes and Genomes (KEGG) [35], and Reactome databases [36]. GSEA was performed using 1000 permutations with weighted enrichment statistics (*p* = 1). Gene set size thresholds were set to 15–500 genes. Significance was determined by FDR < 0.05.

### 2.4. Subtype-Specific Co-Expression Network Analysis and Hub Gene Identification

RNA-seq raw STAR-counts from the TCGA-STAD cohort were used as initial input. Differential expression (DE) analysis between SuStaIn-derived subtypes (AP, *n* = 261; GP, *n* = 143) was performed using DESeq2, incorporating age and gender as covariates in the design formula (~Age + Sex + Subtype). Genes with adjusted *p*-value < 0.05 and |log2 fold change| > 1 were retained. To ensure homoscedasticity for downstream analyses, Variance Stabilizing Transformation (VST) was applied to raw counts prior to all correlation-based analyses, including WGCNA and stage association testing, to ensure consistency with DESeq2 normalization assumptions.

Weighted gene co-expression network analysis (WGCNA) was conducted separately for each subtype using signed networks. Data quality was assessed with the goodSamplesGenes function, and soft-thresholding power was selected using pickSoftThreshold to approximate scale-free topology (R^2^ > 0.9, or power = 6 when unmet). The minimum module size was set to 30 or one-third of the gene number, and modules were merged at a cut height of 0.25. Hub genes were identified using the cytoHubba plugin in Cytoscape (version 3.10.2; Cytoscape Consortium, San Diego, CA, USA) based on Maximal Clique Centrality (MCC), with the top 10 genes retained for each subtype. To capture evolutionary dynamics, Spearman correlations between VST-normalized gene expression and SuStaIn-inferred stage labels were calculated. To minimize false positives from high-dimensional testing, all nominal *p*-values were adjusted using the Benjamini–Hochberg (FDR) method. Stage-associated genes were rigorously defined as those meeting the criteria of ρ > 0.35 and FDR < 0.05. Gene lists with adjusted statistics are provided in Supplementary Table S3.

### 2.5. Squamousness and Gastric Glandular Scoring

To quantify epithelial phenotypic states associated with evolutionary trajectories, Squamousness Score and Gastric Glandular Score were calculated using single-sample gene set enrichment analysis (ssGSEA). Squamousness was defined by the expression of keratinization- and squamous differentiation-related genes (*KRT14*, *PKP1*, *SPRR3*, *SPRR1B*, *SPRR2E*) [37], while gastric glandular identity was represented by gastric lineage markers (*PGA3*, *PGA4*, *PGC*, *GIF*, *ATP4A*, *ATP4B*) [38]. ssGSEA scores were computed on TCGA-STAD RNA-seq data using default parameters, and scores were subsequently correlated with SuStaIn-inferred evolutionary stage labels using Spearman correlation analysis to assess trajectory-dependent phenotypic shifts.

### 2.6. Tumor Microenvironment (TME) Analysis

The tumor microenvironment (TME) composition in late-stage TCGA-STAD samples was analyzed using the EcoTyper framework [39]. EcoTyper was applied to quantify cell populations, including cancer-associated fibroblasts (CAFs), immune cells, and epithelial cells, using transcriptome deconvolution and single-cell RNA sequencing integration. Samples were stratified by subtype as defined by SuStaIn. EcoTyper analysis was performed using log2(TPM + 1) normalized expression data with default deconvolution parameters based on reference cell state signatures.

CAF Abundance: Single-sample gene set enrichment analysis (ssGSEA) was performed using four CAF marker gene sets (Supplementary Table S2) to quantify CAF abundance. The ssGSEA algorithm was implemented with default parameters, and statistical comparisons between subtypes were conducted using the Wilcoxon rank-sum test (*p* < 0.05).

Single-Cell RNA Sequencing: Single-cell RNA sequencing (scRNA-seq) data from a previously published study were utilized [40]. Subclustering was performed to identify fibroblast, endothelial, epithelial, and T-cell subpopulations. Survival associations were evaluated using Cox regression with a significance threshold of *p* < 0.05. Intercellular interactions were assessed using Spearman correlation analysis, with highly significant Spearman correlations (FDR < 0.001), reflecting the robust co-occurrence of cell states within the identified Ecotypes.

### 2.7. Cell–Cell Communication Analysis Between eCAFs and AP-like Epithelial Cells

To investigate stromal–epithelial interactions underlying squamous-like transitions along the AP trajectory, single-cell RNA-seq data from the external cohort GSE183904 [41] were analyzed. Epithelial cells were scored using AUCell [42] based on AP and GP signature gene sets derived from TCGA bulk data and classified as AP-like or GP-like accordingly. Cell–cell communication analysis was performed using CellChat [43] to infer ligand–receptor interactions between eCAFs and epithelial subpopulations. Default CellChat parameters were applied to identify significantly active signaling pathways and ligand–receptor pairs. Ligand specificity was assessed by comparing expression levels of key signaling molecules between eCAFs and other fibroblast populations. To evaluate trajectory-dependent receptor sensitization, expression of epithelial receptors was correlated with SuStaIn-inferred evolutionary stage labels using Spearman correlation analysis. All analyses were conducted using R with standard CellChat and AUCell workflows.

AUCell scoring was performed using the top 5% ranked genes per cell for AUC calculation.

CellChat analysis used the default human ligand–receptor database, with communication probability inferred using the truncated mean method (trim = 0.1) and significance assessed via permutation testing (*n* = 100, *p* < 0.05).

### 2.8. External Validation of AP and GP Evolutionary Trajectories

To validate the AP and GP evolutionary trajectories across independent datasets, we applied a gene signature-based strategy to both bulk and single-cell transcriptomic cohorts. AP and GP signature gene sets were derived from TCGA-STAD based on subtype-specific features and evolutionary associations (Supplementary Table S4). In bulk datasets, including GSE57303 [44], GSE62254 (ACRG) [7], and GSE84437 [45], samples were scored using these signatures and classified as AP-like or GP-like, followed by survival analysis using Kaplan–Meier estimation and the log-rank test. In single-cell RNA-seq datasets, epithelial cells were scored using AUCell, enabling classification into AP-like and GP-like states at single-cell resolution. Across all validation cohorts, AP-like tumors consistently exhibited inferior survival outcomes, confirming the robustness and generalizability of mutation-defined evolutionary trajectories. All gene signatures used for validation were predefined in the TCGA cohort and applied unchanged to external datasets.

### 2.9. Validation of Mutation Progression Sequences Using TRONCO

Mutation progression sequences inferred by SuStaIn were validated using the TRONCO R package (version 2.40.0) (TRONCO Bioconductor) [46], which employs the Suppes-Bayes Causal Network approach to infer cancer progression models. Mutation matrices for Subtype 1 (AP, *n* = 260) and Subtype 2 (GP, *n* = 140) were constructed, with rows representing tumor samples and columns indicating the cumulative frequency of mutations in selected genes and gene families. TRONCO was configured with default parameters for cross-sectional data, generating subtype-specific evolutionary trees. These trees were converted into ordered gene family pairs, which were compared with SuStaIn-derived mutation sequences to quantify concordance. All analyses were performed in R (version 4.4.1).

The mutation progression sequences inferred by SuStaIn for Subtype 1 (AP, *n* = 260) and Subtype 2 (GP, *n* = 140) were validated using the TRONCO R package (version 2.40.0) [40], which implements the Suppes-Bayes Causal Network approach to model cancer progression. For each subtype, SuStaIn derived a linear sequence of mutation events, based on mutation data from TCGA. Using TRONCO for cross-sectional data, subtype-specific evolutionary trees were generated, where nodes represent gene families and edges indicate progression relationships. To compare the linear SuStaIn sequences with the tree-based TRONCO models, all possible gene family pairs were extracted from the gene lists (identical across both methods). For each gene family pair, the order of appearance in the SuStaIn sequence was compared to the order implied by the TRONCO tree (based on parent–child relationships). The concordance rate was calculated as the proportion of gene pairs with consistent ordering between the two methods (i.e., number of pairs with matching order divided by total gene pairs). All analyses were performed in R (version 4.4.2).

### 2.10. Drug Sensitivity Prediction and Trajectory-Associated Analysis

Drug sensitivity was inferred using the oncoPredict [47] framework based on ridge regression models trained on the GDSC2 dataset. Preprocessed GDSC2 expression profiles (RMA-normalized and log-transformed) and corresponding drug response data were used as the training reference. TCGA-STAD bulk RNA-seq data were log2-transformed (TPM + 1) and batch-corrected using standardization prior to prediction. Predicted drug response was represented as ln(IC50) values for cisplatin, 5-fluorouracil, and docetaxel. Samples were annotated with subtype labels and SuStaIn-inferred evolutionary stage indices. Associations between predicted ln(IC50) values and evolutionary stages were assessed using Spearman correlation analysis, both globally and stratified by subtype. Differences in correlation strength between subtypes were evaluated using Fisher’s z-transformation. Temporal trends in predicted drug sensitivity were visualized using scatter plots with linear regression fits. All analyses were performed in R. Ridge regression models were trained using 10-fold cross-validation in the GDSC2 dataset. Gene matching between datasets was restricted to shared genes, and expression values were standardized prior to prediction.

### 2.11. Statistical Analysis

All statistical analyses were performed using R (version 4.4.3) and Python (version 3.11). Unless otherwise specified, all tests were two-sided, and a *p*-value < 0.05 was considered statistically significant. For high-dimensional data, multiple testing correction was conducted using the Benjamini–Hochberg method, and false discovery rate (FDR)-adjusted *p*-values were reported where applicable.

Survival differences between groups were evaluated using Kaplan–Meier analysis and compared using the log-rank test. Clinical characteristics were compared using the Chi-square test or Fisher’s exact test, as appropriate. Differences in tumor microenvironment (TME) cell populations between groups were assessed using the Wilcoxon rank-sum test.

Correlation analyses were performed using Spearman rank correlation. For genome-wide correlation analyses, *p*-values were adjusted using the Benjamini–Hochberg method to control for multiple comparisons. Where applicable, differences between correlation coefficients were assessed using Fisher’s z-transformation.

## 3. Results

### 3.1. SuStaIn Reveals Two Distinct Mutational Trajectories in Gastric Cancer

To model the temporal sequence of somatic mutation accumulation in gastric cancer (GC), we employed the Subtype and Stage Inference (SuStaIn) algorithm to reconstruct the temporal sequence of somatic mutation accumulation. Analysis of TCGA-STAD profiles identified two distinct mutational ordering patterns: Subtype 1 (*n* = 260, 65%) and Subtype 2 (*n* = 140, 35%), designated as the Accelerated Path (AP) and Gradual Path (GP) based on the relative timing of driver events. The robustness of this stratification was supported by high mean posterior probabilities for both subtype (P_subtype_ = 0.947) and stage (P_stage_ = 0.935) assignments (Supplementary Table S6). The AP and GP were primarily distinguished by the inferred ordering of key driver events rather than the total mutational burden at the clinical endpoint (*p* > 0.05, Supplementary Table S7). Notably, the model positioned the pivotal tumor suppressor *TP53* at a significantly earlier pseudotime stage in the AP (Stage 3), whereas it appeared later in the inferred sequence for the GP (Stage 8). This difference in ordering indicates that in AP tumors, canonical driver mutations occur earlier in the inferred sequence, whereas in GP tumors, these events are deferred to later stages in the model.

**Figure 2:**
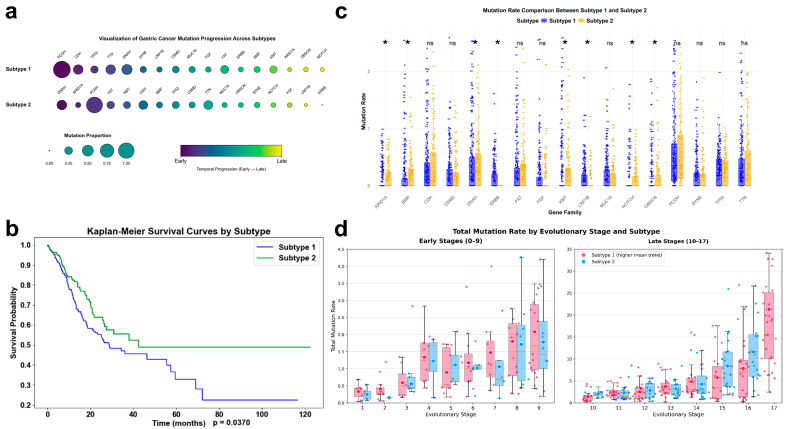
Somatic mutation trajectories define prognostically distinct gastric cancer subtypes. (**a**) Evolutionary mutation patterns across gastric cancer subtypes. Scatter plot depicting subtype-specific somatic mutation trajectories inferred by SuStaIn. Each dot represents a gene-specific mutation event, ordered from left to right according to inferred evolutionary stage. Colors indicate progression stages, while dot size reflects mutation frequency within each subtype, illustrating distinct temporal accumulation patterns between Subtype 1(AP) and Subtype 2(GP). (**b**) Overall survival stratified by mutation-defined subtypes. Kaplan–Meier curves comparing overall survival between Subtype 1 and Subtype 2. Statistical significance was assessed using the log-rank test. (**c**) Comparison of mutation burden across subtypes. Boxplots with overlaid individual samples showing subtype-specific mutation rates across major gene families. Statistical significance is indicated (* *p* < 0.05; ns, not significant). (**d**) Stage-wise comparison of cumulative mutation rates between subtypes. Boxplots comparing the summed mutation rates at each inferred evolutionary stage between Subtype 1(AP) and Subtype 2(GP). The left panel shows early stages (Stages 1–9), while the right panel displays later stages (Stages 10–17) to accommodate differences in scale. Colored markers indicate the mean mutation rate for each stage. Subtype 1 exhibits consistently higher average mutation rates during early evolutionary stages, indicating accelerated mutational accumulation at tumor initiation.

Clinically, these trajectory assignments were associated with markedly different outcomes. Kaplan–Meier analysis revealed that patients in the AP group had significantly worse overall survival (OS) compared to those in the GP group (median OS: 13.43 vs. 18.38 months; log-rank *p* = 0.0370) (Figure 2b). To evaluate the independent prognostic value of this classification, we performed a multivariable Cox proportional hazards analysis. After adjusting for age, AJCC pathologic stage (Supplementary Table S9; all |r| < 0.1), and molecular subtypes (MSI and EBV status), the AP assignment remained an independent predictor of worse OS (Hazard Ratio = 1.437, 95% CI: 1.009–2.047, *p* = 0.044; Table 1).

Furthermore, the AP was enriched with high-risk clinical features, including a significantly higher proportion of N3 stage lymph node metastasis (24.2% vs. 13.6%, *p* < 0.05, Table 2), underscoring the clinical relevance of this inferred trajectory. Statistical comparison of the mutational sequences highlighted the divergence between the two paths. The Kendall’s Tau (0.176) and Spearman correlation (0.213) between the AP and GP sequences were notably low. The Longest Common Subsequence (LCS) included only seven genes (*PCDH, CDH, TP53, CSMD, MUC16, OBSCN, NOTCH*), consistent with the model inference that the temporal logic of mutation accumulation differs by SuStaIn subtype. Notably, ERBB family mutations (specifically *ERBB3* and *ERBB4*) were exclusively detected among the later-stage events within the AP.

Finally, the stability of these two identified ordering patterns (AP and GP) was further validated through a leave-out sensitivity analysis, where the exclusion of hyper-mutated large genes did not alter the inferred temporal sequence of oncogenic events.

### 3.2. Transcriptomic Features Associated with Inferred Mutational Timing in AP and GP Subtypes

**Figure 3:**
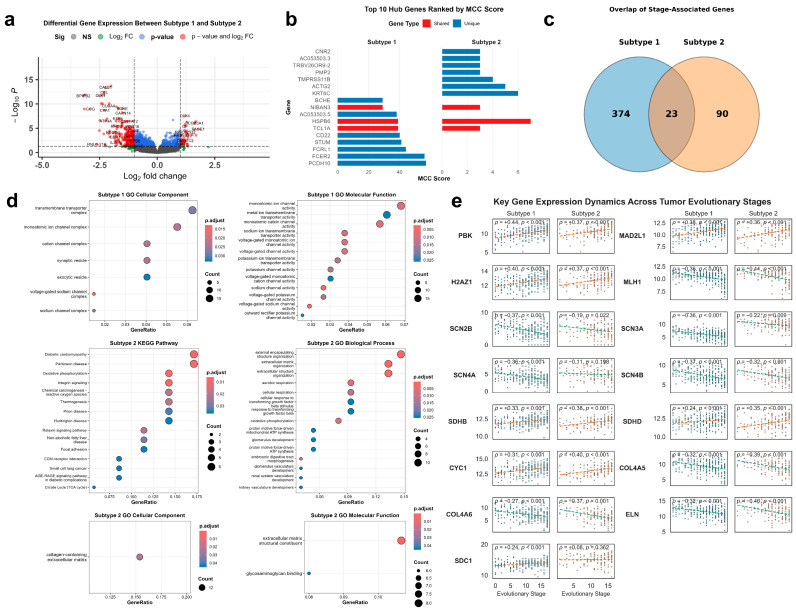
Transcriptomic programs and network-level regulators associated with evolutionary subtypes. (**a**) Differential gene expression between gastric cancer subtypes. Volcano plot illustrating genes differentially expressed between Subtype 1(AP) and Subtype 2(GP). Gray dots represent non-significant genes. Blue dots indicate genes significant only by adjusted *p*-value (but not by fold change). Green dots indicate genes significant only by |log_2_FC| (but not by adjusted *p*-value). Red dots indicate genes significant by both criteria. The black dashed lines denote the significance thresholds: adjusted *p*-value < 0.05 and |log_2_FC| > 1. (**b**) Identification of subtype-associated hub genes by WGCNA. Top 10 hub genes for each subtype identified from weighted gene co-expression network analysis (WGCNA), ranked by Maximal Clique Centrality (MCC) scores, indicating their regulatory importance within subtype-specific modules. (**c**) Overlap of stage-associated genes between gastric cancer subtypes. Venn diagram showing genes significantly correlated with inferred evolutionary stages in Subtype 1 and Subtype 2 (|ρ| > 0.35, *p* < 0.05). Twenty-three genes were shared by both subtypes, while 374 and 90 genes were uniquely associated with Subtype 1 and Subtype 2, respectively. (**d**) Functional enrichment of subtype-associated genes. Gene Ontology (biological process, molecular function, cellular component) and KEGG pathway enrichment analyses of differentially expressed genes, revealing subtype-specific biological programs. (**e**) Temporal dynamics of key genes along evolutionary trajectories. > Representative subtype-associated genes showing expression changes across inferred SuStaIn stages. Linear regression lines (dashed) illustrate trends along the temporal axis. Spearman correlation coefficients (ρ) and Benjamini–Hochberg adjusted *p*-values (q) are annotated. The robust association of *SCN4A*, *SDHD*, and *PBK* with evolutionary pseudo-time (q < 0.001) links transcriptional reprogramming to mutation-driven progression.

To identify transcriptomic patterns associated with the inferred temporal progression in each SuStaIn subtype, we evaluated the correlation between pseudotime stages (1–17) and gene expression profiles. We identified a set of 23 genes that exhibited consistent correlation with stage across both subtypes (all ρ > 0.35, FDR < 0.05). This shared set included progressive upregulation of cell cycle regulators, such as *PBK* (FDR_AP_ = 1.28 × 10^−10^, FDR_GP_ = 7.35 × 10^−4^), *MAD2L1*, and *H2AZ1*, alongside decreased expression of the DNA mismatch repair gene *MLH1* (Figure 3e). These data indicate an association between later pseudotime stages and both increased proliferative gene expression and reduced expression of repair machinery in both subtypes.

**Figure 4:**
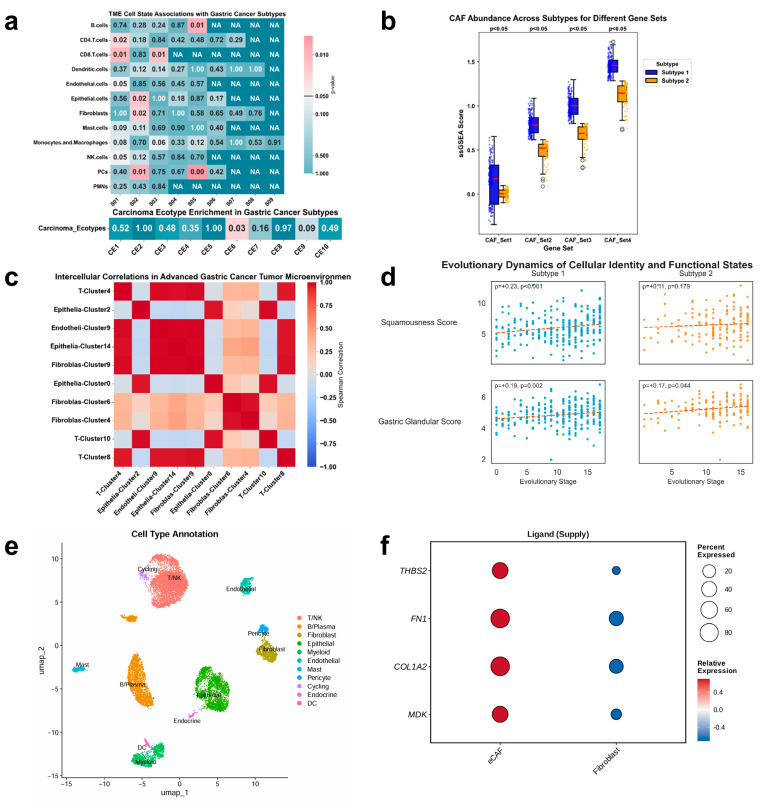
Evolutionary subtypes shape distinct tumor microenvironment landscapes. (**a**) Association between gastric cancer subtypes and tumor microenvironment states. Heatmap displaying chi-square test *p*-values for associations between cancer epithelial states (CE1–CE10), non-malignant cell states (S01–S09), and gastric cancer subtypes. Significant associations (*p* < 0.05) are highlighted, indicating subtype-specific TME enrichment patterns. (**b**) Enrichment of cancer-associated fibroblasts in Subtype 1. Boxplots with individual samples showing ssGSEA scores for four CAF gene signatures across subtypes. Subtype 1 exhibits significantly higher CAF abundance (*p* < 0.05). The red horizontal line inside each box indicates the median. (**c**) Cell–cell coordination within advanced gastric cancer TME. Spearman correlation heatmap illustrating interactions among fibroblast, epithelial, endothelial, and T-cell subpopulations. Strong correlations (>0.96) suggest coordinated remodeling of the tumor microenvironment. (**d**) Temporal evolution of phenotypic scores along mutation trajectories. Changes in Squamousness Score and Gastric Glandular Score across inferred evolutionary stages in Subtype 1 and Subtype 2. Linear regression and Spearman correlation analyses reveal subtype-specific phenotypic shifts driven by mutation accumulation. The dashed straight lines in the figure represent the fitted trends of phenotypic scores as evolutionary stages progress. (**e**) Single-cell landscape of the external validation cohort (GSE183904). UMAP projection of single-cell RNA-seq data from GSE183904, with cells colored by annotated cell types. Major epithelial, stromal, and immune populations are labeled, providing an independent single-cell framework for validating subtype-associated stromal–epithelial interactions. (**f**) Enrichment of eCAF-derived signaling factors targeting AP epithelial cells. Comparison of expression levels of *MDK, THBS2, COL1A2,* and *FN1* between eCAFs and other fibroblast populations. Boxplots show significantly higher expression of all four ligands in eCAFs, supporting an eCAF-dominant source of neurotrophic and ECM-mediated signaling implicated in AP-specific stromal–epithelial communication.

In addition to the shared features, stage-correlated expression in the AP subtype exhibited distinct characteristics. The voltage-gated sodium channel gene *SCN4A* showed a significant negative correlation with AP stage (ρ = −0.36, FDR = 1.18 × 10^−7^), whereas this relationship was not observed in the GP subtype (ρ = −0.11, *p* = 0.198). This pattern, along with similar trends among other sodium channel complex genes (*SCN2B, SCN3A, SCN4B*), suggests that AP stage progression is correlated with altered expression of ion transport pathways. Furthermore, progression along the AP stages was accompanied by a specific increase in the Squamous Score (Spearman r = 0.23, *p* < 0.001), with no significant trend observed in the GP (r = 0.11, *p* = 0.179) (Figure 4d). While the simultaneous changes in ion channel expression and squamous-associated gene signatures may indicate phenotypic divergence, the current analysis only establishes a correlative link between inferred mutational order and these bulk transcriptional features.

In contrast, the GP subtype displayed a more pronounced correlation between stage and maintained expression of oxidative phosphorylation components. The mitochondrial complex II subunit *SDHD* was negatively correlated with GP stage (ρ = −0.35, FDR = 1.01 × 10^−3^). These stage-associated expression patterns differ notably from those seen in the AP subtype.

### 3.3. Tumor Microenvironment Features Associated with the AP and GP Subtypes

Given the association between mutational order and clinical outcome, we next examined the tumor microenvironment (TME) composition across the two inferred trajectories. Using EcoTyper and single-cell RNA sequencing (scRNA-seq) deconvolution, we observed that the AP (Subtype 1) was associated with lower inferred immune infiltration scores and higher stromal signatures relative to the GP (Subtype 2) (Figure 4b).

Specifically, gene signatures corresponding to Cancer-Associated Fibroblasts (CAFs) and endothelial cells were more highly expressed in AP tumors. Among these, single-cell sub-clustering identified two fibroblast populations (Fibroblast-Cluster 4 and 6) with transcriptomic profiles consistent with extracellular matrix CAFs (eCAFs). The inferred abundance of these eCAF populations exhibited a high correlation with the abundance of AP-like epithelial cell states across samples (Spearman r > 0.96). While these correlations do not establish causality, they indicate a non-random association between the inferred AP trajectory and stromal cell abundance estimates.

To identify potential molecular interactions that may underlie this association, we performed cell–cell communication analysis using CellChat. This analysis predicted that eCAFs express higher levels of several ligands—including *Midkine (MDK), THBS2, COL1A2,* and *FN1*—relative to other fibroblast populations (Figure 4f). Ligand-specific expression comparison confirmed that *THBS2* and *MDK* transcripts were, on average, 24.2-fold and 7.0-fold higher in eCAFs compared to regular fibroblasts, respectively. Furthermore, the receptor *SDC1*, although detected in both subtypes, showed a significant positive correlation with AP pseudotime stages (ρ = 0.24, *p* < 0.001), which was not observed in the GP subtype (ρ = 0.08, *p* = 0.362) (Figure 3e). These data suggest a pattern whereby AP stage progression is coupled with increased *SDC1* expression, which may influence the tumor’s responsiveness to eCAF-derived ligands; however, functional validation would be required to test this hypothesis.

In addition, the AP subtype was characterized by lower inferred abundance of CD8^+^ S01 T cells compared to the GP subtype. Conversely, the GP subtype exhibited higher inferred signatures for activated B cells and effector memory T cells.

### 3.4. Validation of Subtype Classification and Reproducibility Across External Cohorts

**Figure 5:**
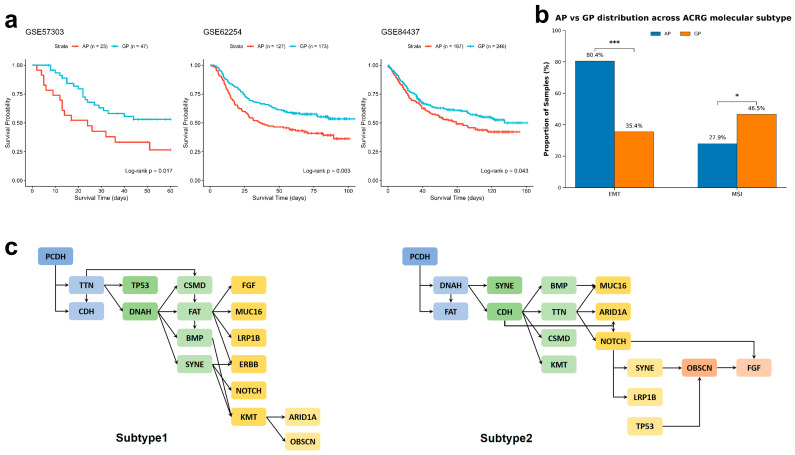
Cross-cohort validation of AP/GP trajectories and their clinical relevance. (**a**) Prognostic validation of AP and GP trajectories across independent bulk cohorts. Kaplan–Meier survival analyses stratified by AP-like and GP-like classification in three independent gastric cancer cohorts (GSE57303, GSE62254/ACRG, and GSE84437). Samples were assigned based on enrichment of AP and GP signature gene sets derived from TCGA. Across all cohorts, AP-like tumors consistently exhibited significantly poorer overall survival compared with GP-like tumors, confirming the robustness and prognostic relevance of the evolutionary trajectories. (**b**) Distribution of ACRG molecular subtypes within AP-like and GP-like tumors in the GSE62254 cohort. AP-like tumors were significantly enriched for the EMT subtype (80.4% vs. 35.4%, *p* < 0.001), whereas GP-like tumors showed higher representation of the MSI subtype (46.5% vs. 27.9%, *p* = 0.031). These results indicate a statistical association between the inferred mutational trajectories and ACRG subtypes, consistent with the higher stromal and CAF-related signatures observed in AP tumors. Data are presented as percentages of samples in each group. Statistical significance was assessed using Fisher’s exact test with Bonferroni correction. * *p* < 0.05, *** *p* < 0.001. (**c**) Subtype-specific evolutionary trees inferred by TRONCO. Progression models reconstructed for Subtype 1 and Subtype 2, illustrating distinct mutational dependencies and evolutionary paths, consistent with SuStaIn-derived trajectories.

To assess whether the AP and GP signatures were associated with clinical outcomes in independent datasets, we applied the gene signatures derived from our SuStaIn-correlated analysis—which include the *SCN4A*/*SCN*-family and *SDHD* genes—to three independent transcriptomic datasets (GSE57303, GSE62254/ACRG, and GSE84437). In all three cohorts, tumors classified as AP-like exhibited significantly shorter overall survival compared to GP-like tumors (*p* = 0.0166, *p* = 0.0033, and *p* = 0.0428, respectively; Figure 5a). This consistent survival difference supports the prognostic relevance of the AP/GP signature-based classification across independent populations.

Next, we integrated our classification with the ACRG (GSE62254) molecular subtyping system. A significantly higher proportion of AP-like tumors were classified as the EMT (Epithelial-to-Mesenchymal Transition) subtype (80.4%) compared to GP-like tumors (35.4%) (*p* < 0.001; Figure 5b). This enrichment is consistent with the increased stromal and CAF-related signatures we observed in the AP subtype. Conversely, the GP subtype was enriched for the MSI (Microsatellite Instability) subtype (46.5% vs. 27.9%, *p* = 0.031), in line with the higher inferred immune cell signatures observed in this group. Furthermore, mutational trees independently reconstructed by TRONCO showed a 67.9% concordance with the AP SuStaIn sequence (Figure 5c).

Collectively, the external validations show that the AP and GP subtypes—defined by mutational ordering patterns—are consistently associated with survival differences, distinct molecular subtype enrichments, and compatible mutational tree structures across multiple independent cohorts, supporting the reproducibility of this classification.

### 3.5. Association of Inferred Chemosensitivity with AP Evolutionary Stage

**Figure 6:**
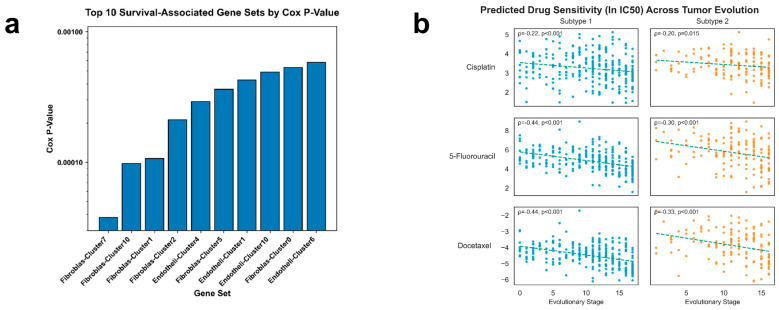
Clinical relevance of evolutionary trajectories: survival programs and therapeutic sensitivity. (**a**) Survival-associated gene programs derived from single-cell data. Top 10 gene sets originating from single-cell RNA-sequencing analyses ranked by Cox proportional hazards *p*-values, highlighting cell-type-specific programs linked to patient survival. (**b**) Evolutionary dynamics of chemotherapy sensitivity. Predicted IC50 values for Cisplatin, 5-Fluorouracil, and Docetaxel plotted against inferred evolutionary stages for Subtype 1 and Subtype 2. Linear regression trends and Spearman correlations demonstrate stage-dependent changes in drug sensitivity. The dashed straight lines in the figure represent the fitted trends of chemotherapy sensitivity as evolutionary stages progress.

Finally, we examined whether the inferred evolutionary stage correlated with predicted chemotherapeutic sensitivity. In the AP subtype, the predicted IC_50_ values for 5-Fluorouracil (5-FU) and Docetaxel showed statistically significant negative correlations with evolutionary stage (ρ = −0.439 and ρ = −0.436, respectively, *p* < 0.001), indicating that tumors at later AP stages had lower predicted IC_50_ estimates in this analysis (Figure 6b). These correlations were accompanied by stage-associated increases in cell cycle gene expression (e.g., *PBK*) and stage-associated changes in *SCN* family ion channel expression.

In the GP subtype, the correlations between stage and predicted 5-FU or Docetaxel sensitivity were weaker, though predicted Cisplatin sensitivity showed similar trends across both subtypes. These results suggest that, within the AP classification, later pseudotime stage is associated with higher predicted sensitivity to 5-FU and Docetaxel in computational models. Whether this statistical association reflects actionable therapeutic vulnerability requires experimental validation.

## 4. Discussion

### 4.1. Inferred Mutational Timing Patterns and Their Divergence

The Lauren and TCGA classification systems have provided a valuable framework for characterizing the molecular features of gastric cancer (GC), yet they largely describe the disease at a single cross-sectional time point. In this study, we applied the SuStaIn algorithm to reconstruct the temporal sequence of somatic mutation accumulation, which allowed us to explore how mutational events may be ordered along a temporal axis. Our analysis identified two distinct mutational trajectories, which we termed the Accelerated Path (AP) and Gradual Path (GP) based on the relative timing of driver events. These two patterns suggest that the inferred order of mutations, rather than a single linear progression, can vary across tumors. Notably, the model positioned *TP53* mutations at early evolutionary stages in the AP and later stages in the GP, raising the possibility that the timing of *TP53* loss may represent one of the early differences between the two subtypes.

The AP and GP trajectories differ primarily in the inferred temporal placement of canonical driver mutations, rather than in the total number of mutations at the clinical endpoint. In the AP, *TP53* was assigned to an early inferred stage, whereas in the GP it appeared later. This early placement was followed in the model by a more rapid succession of subsequent driver events, suggesting that, in AP tumors, key mutations tend to be concentrated toward the beginning of the inferred sequence. One possible interpretation is that early *TP53* mutations are associated with a higher subsequent rate of detectable genomic changes; however, the current cross-sectional data and modeling approach do not directly measure genomic instability rates in real time. The detection of *ERBB* family mutations exclusively in AP tumors further supports the idea that these subtypes are associated with distinct mutational profiles, although the functional consequences of this association remain to be investigated. In the GP, *TP53* mutations were inferred at Stage 8, substantially later than in the AP, and other driver events also appeared later in the sequence, consistent with a more evenly distributed accumulation of mutations across stages. Overall, these differences in the inferred ordering patterns may help explain why tumors with similar final mutational burdens can exhibit divergent clinical behaviors—they appear to follow distinct temporal sequences of mutation accumulation, as estimated by the SuStaIn model.

### 4.2. Bioelectric Signaling Patterns and Their Association with AP Stage Progression

One feature observed in the AP subtype was the stage-correlated downregulation of the voltage-gated sodium channel gene *SCN4A* (ρ = −0.36, *p* < 0.001), a pattern that was not detected in the GP subtype. *SCN4A* has been reported in previous studies to be associated with cancer progression in several malignancies; for instance, its upregulation has been linked to high-risk colorectal cancer groups, and in melanoma, wild-type *SCN4A* has been suggested to impair anti-tumor immune responses [48,49]. In our analysis, *SCN4A* expression declined with advancing AP stage, indicating a statistical association between its transcriptional levels and the inferred mutation order in this subtype. Whether this expression change reflects alterations in ion transport or membrane potential cannot be determined from the current transcriptomic data alone.

We also explored whether the AP-associated TME features might be related to the *SCN4A* pattern. The CellChat analysis identified an interaction axis between eCAF-derived *Midkine (MDK)* and the *SDC1* receptor, the latter showing a positive correlation with AP stage. *MDK* is known as a neurotrophic growth factor, and previous literature has described its involvement in tumor-stroma communication [50,51,52,53]. The co-occurrence of declining *SCN4A* expression, rising *SDC1* expression, and the inferred abundance of eCAFs in AP tumors raises the possibility that these features are jointly associated with AP stage progression; however, the current analysis establishes only correlative links and does not demonstrate a coordinated biological program or a “neural-like” cellular state.

The parallel increase in the Squamous Score along AP stages further distinguishes the AP from the GP trajectory in terms of bulk transcriptional profiles. We emphasize that the squamous-associated gene signature used here provides an indirect, transcriptome-based measure. Cellular identity cannot be definitively inferred from these scores. Given the heterogeneity of gastric cancer and the absence of systematic cross-validation with histopathology or Lauren classification in this study, these transcriptional patterns should be interpreted as hypothesized phenotypic associations rather than as evidence of a lineage transition or a “neural-squamous” cell state. Validation with broader, pan-cancer-validated gene signatures and expert pathological review is required to determine the extent to which these transcriptomic differences reflect histological dedifferentiation along the AP trajectory.

### 4.3. Mitochondrial Gene Expression Patterns Associated with the GP Subtype

In contrast to the AP, the GP subtype exhibited a stage-correlated decrease in the expression of the mitochondrial complex II subunit *SDHD* (ρ = −0.35, *p* < 0.001). As a component of both the TCA cycle and the electron transport chain, *SDHD* encodes a protein involved in oxidative phosphorylation (OXPHOS) [54]. The observed correlation between GP stage and *SDHD* expression suggests an association between the inferred mutational order in this subtype and the transcriptional output of mitochondrial pathways.

Previous studies have reported that *SDHD* depletion can promote thyroid tumorigenesis and that *SDHD* alterations have been implicated in gastrointestinal malignancies through disruption of mitochondrial respiratory chain functions [55,56]. In our data, *SDHD* levels declined with advancing GP stage, a pattern that differs from that observed in the AP. One possible interpretation is that, relative to the AP, the GP is associated with a more gradual change in OXPHOS-related gene expression across evolutionary stages; however, the current analysis does not directly measure mitochondrial function or metabolic flux.

GP tumors were also associated with higher inferred scores for activated B cells and effector memory T cells, along with more stable gastric identity scores. Whether this statistical pattern reflects a distinct metabolic or immunological interaction cannot be determined from the present data. The divergence between the *SCN4A*-associated expression patterns in the AP and the *SDHD*-associated expression patterns in the GP thus provides a transcriptomic framework for further investigation into the molecular differences between these two subtypes. Functional studies will be required to test any mechanistic link between these gene expression patterns and the divergent clinical outcomes observed.

### 4.4. Associations Between Inferred Tumor-Stroma Interactions and AP Stage Progression

Our analysis integrated transcriptomic profiles from bulk and single-cell data with the inferred mutational trajectories to examine patterns of tumor–stroma association. In AP tumors, the inferred abundance of eCAF populations was highly correlated with that of AP-like epithelial cells across samples (Spearman r > 0.96). CellChat analysis further identified predicted ligand–receptor interactions between these compartments, including MDK–SDC1 and THBS2-related pathways [57,58]. *SDC1* expression increased with AP stage, and *MDK* and *THBS2* were predicted to be more highly expressed in eCAFs than in other fibroblast populations. These observations indicate that expression of these ligands and receptor components co-varies with AP stage and inferred stromal abundance, but they do not establish a directional or causal signaling program.

Previous studies have associated CAF-derived extracellular matrix (ECM) components with both structural remodeling and modulation of immune infiltration [1,59,60]. In our data, AP tumors were characterized by lower inferred CD8^+^ T-cell scores and higher stromal signatures, which is consistent with an immune-excluded pattern. However, whether this pattern results from physical ECM barriers, chemokine gradients, or other mechanisms cannot be distinguished from transcriptomic data alone.

The high correlation between stromal and epithelial cell state scores, together with the coordinated appearance of ligand–receptor pairs in the inferred trajectories, is compatible with the hypothesis that the AP mutational order and the associated stromal changes occur in a non-random, temporally coordinated manner. However, this coordination is inferred from cross-sectional samples and does not demonstrate functional interdependence.

We therefore emphasize that the tumor microenvironment associations reported here are derived from computational deconvolution and inferred cell–cell communication analysis. The absence of spatial transcriptomics or direct functional perturbation leaves open the question of causal direction. The proposed relationship between the AP mutational sequence, cell-extrinsic signaling, and the transcriptomic patterns described above should be viewed as a hypothesis-generating framework. Future studies using spatial proteogenomic approaches and experimental validation will be necessary to determine whether these inferred interactions are causally related to the transcriptional phenotypes we observed along the AP trajectory.

### 4.5. Methodological Innovation: Use of a Discrete Event-Based Model to Infer Mutational Sequences

A significant innovation of this study lies in the application of the Subtype and Stage Inference (SuStaIn) algorithm [25,26] to reconstruct the temporal sequence of somatic mutation accumulation. Unlike single-cell transcriptomic trajectory inference methods (e.g., Monocle, Slingshot) that infer continuous gene expression paths, SuStaIn is based on a discrete event-based modeling framework and was developed to order categorical events (here, the presence of somatic mutations) along a disease progression axis. While transcriptomic trajectory methods can capture gradual expression changes, they are sensitive to technical noise and are not designed to represent somatic mutations as irreversible discrete events. SuStaIn models the accumulation of somatic mutations as an ordered sequence of discrete events.

Using a Bayesian framework and MCMC sampling, the algorithm deconvolves population-level heterogeneity into distinct trajectories (AP and GP) and simultaneously estimates the most probable sequence of events without requiring predefined stage labels. To our knowledge, this is the first application of an event-based disease progression model—previously used primarily in imaging-based staging of neurodegenerative disorders—to somatic mutation data in gastric cancer. This approach yields an inferred ordering of mutational events, which we show is associated with clinical outcome differences and is reproducible across independent cohorts.

Rather than providing a continuous cell-state manifold, the SuStaIn output offers a discrete sequence that can be directly related to the observed mutational data. This provides a complementary perspective to static molecular classifications by ordering the accumulation of mutations along an inferred temporal axis.

### 4.6. Clinical and Translational Correlates of the AP and GP Trajectories

Our analysis identified a statistically significant negative correlation between inferred AP evolutionary stage and predicted IC_50_ values for Docetaxel and 5-Fluorouracil (5-FU) using oncoPredict [61]. This indicates that, in this computational model, tumors classified as late-stage AP are associated with lower predicted drug sensitivity estimates. One possible explanation is that the stage-associated changes in gene expression observed in the AP—including declining *SCN4A* levels and increasing *PBK* expression—correlate with predicted sensitivity to agents that target proliferating cells in these models [62].

However, we stress that these predictions are derived solely from transcriptome-based IC_50_ estimation and have not been validated in patient-derived models or independent pharmacogenomic cohorts. Accordingly, the observed association should be viewed as a computational pattern requiring experimental confirmation, not as evidence of a clinically actionable therapeutic window.

If validated in future studies, the expression patterns of *SCN4A*, *SDHD*, and associated mutational signatures could be explored as candidate biomarkers for patient stratification in preclinical settings. For GP tumors, which were associated with higher inferred immune cell scores, preclinical investigation of immunotherapy or OXPHOS-targeting strategies may be warranted [63,64,65]. The correlation between *SCN* family ion channel expression and AP stage also raises the possibility—but does not establish—that ion channel activity contributes to the AP-associated transcriptional phenotype; this hypothesis would require direct functional testing before any therapeutic exploration of sodium channel blockers could be considered [66].

Several methodological limitations should be considered when interpreting these findings. First, our external validation was based on transcriptomic signatures rather than on independently reconstructed mutational trajectories, because the external cohorts lacked the longitudinal or multi-regional mutation data required to rerun SuStaIn. Therefore, the validation demonstrates the reproducibility of the AP/GP gene expression patterns across populations, but does not independently replicate the underlying mutation-order model. Second, the partial concordance (67.9%) between SuStaIn and TRONCO-derived mutational trees reflects consistency between two algorithms applied to the same TCGA-STAD dataset, rather than independent validation against a ground-truth temporal sequence. Both methods share the same cross-sectional input, and the agreement observed is therefore best interpreted as inter-algorithm consistency. Definitive validation of the inferred mutational order will require true longitudinal sampling or multi-region sequencing studies, which are not currently available in public gastric cancer datasets.

## 5. Conclusions

In conclusion, this study identifies two distinct mutational ordering patterns in gastric cancer—the Accelerated Path (AP) and the Gradual Path (GP)—which are associated with different transcriptomic features and clinical outcomes. The AP subtype is characterized by stage-correlated downregulation of *SCN4A* and higher stromal and eCAF signatures, whereas the GP subtype is associated with *SDHD*-correlated expression patterns and higher inferred immune cell scores. Computational drug sensitivity predictions suggest an association between later AP stage and lower predicted IC_50_ values for certain chemotherapeutic agents; this observation is a hypothesis-generating finding that requires experimental validation. These results demonstrate that the temporal order of mutation accumulation, as inferred by SuStaIn, is correlated with the molecular and clinical heterogeneity of gastric cancer. Whether these inferred trajectories can inform therapeutic strategies or define periods of increased treatment sensitivity must be evaluated in future functional and longitudinal studies.

## Figures and Tables

**Table 1:** Multivariable Cox proportional hazards model for overall survival in TCGA-STAD.

Covariate	HR (exp(coef))	95% CI for HR	*p*-Value
Evolutionary Subtype (AP vs. GP)	1.437	1.009–2.047	0.044
Age	1.03	1.013–1.047	<0.0005
Pathologic Stage (Advanced)	1.815	1.470–2.240	<0.0005
MSI Status (Positive vs. Negative)	0.55	0.330–0.916	0.022
EBV Status (Positive vs. Negative)	0.957	0.493–1.860	0.897

**Table 2:** The two cancer progression subtypes and patient characteristics.

Characteristics	AP (Subtype 1)	GP (Subtype 2)	*p*-Value
pT stage			
T1	14 (5.4%)	8 (5.7%)	
T2	50 (19.2%)	36 (25.7%)	
T3	128 (49.2%)	60 (42.9%)	
T4	68 (26.2%)	36 (25.7%)	
pN stage			
N0	80 (30.8%)	45 (32.1%)	
N1	68 (26.2%)	43 (30.7%)	
N2	49 (18.8%)	33 (23.6%)	
N3	63 (24.2%)	19 (13.6%)	0.0168
AGE			
<40	2 (0.8%)	2 (1.4%)	
40–60	79 (30.4%)	35 (25.0%)	
60–80	161 (61.9%)	86 (61.4%)	
≥80	18 (6.9%)	17 (12.1%)	
SEX			
Male	165 (63.5%)	95 (67.9%)	
Female	95 (36.5%)	45 (32.1%)	
RACE			
White	171 (65.8%)	77 (55.0%)	0.0445
Asian	51 (19.6%)	34 (24.3%)	
Black or African American	10 (3.8%)	2 (1.4%)	

## Data Availability

The genomic datasets supporting the conclusions of this article are available in the NCBI GEO repository (https://www.ncbi.nlm.nih.gov/geo/) (accessed on 23 April 2025) and The Cancer Genome Atlas (TCGA) database through the UCSC Xena platform (http://xena.ucsc.edu/) (accessed on 24 January 2025).

## Supplementary Materials

The following supporting information can be downloaded at: https://www.mdpi.com/article/10.3390/genes17050536/s1, Supplementary Table S1: Gene-to-family mapping of somatic mutations in gastric cancer. Supplementary Table S2: Marker gene sets for cancer-associated fibroblasts (CAFs) used in ssGSEA analysis [67,68,69]. Supplementary Table S3. Stage-associated genes correlated with SuStaIn-inferred evolutionary progression in AP and GP subtypes. Supplementary Table S4. AP and GP signature gene sets derived from TCGA-STAD for cross-cohort and single-cell validation. Supplementary Table S5. Sensitivity analysis of SuStaIn model robustness after excluding hyper-mutated large genes (TTN and MUC16). Supplementary Table S6. Assessment of model assignment confidence and temporal uncertainty. Supplementary Table S7. Comparison of pan-cohort genomic features between AP and GP subtypes. Supplementary Table S8. Distribution of AP and GP subtypes across TCGA molecular classifications. Supplementary Table S9. Module-Trait correlations with AJCC Staging.

## Abbreviations

TCM	traditional chinese medicine
TCGA	the cancer genome atlas
GEO	gene expression omnibus
FDR	false discovery rate
FC	fold change
GSEA	gene set enrichment analysis
ssGSEA	single-sample gene set enrichment analysis
DEGs	differentially expressed genes
TME	tumor microenvironment
SuStaIn	Subtype and Stage Inference
CAFs	cancer-associated fibroblasts
KEGG	Kyoto Encyclopedia of Genes and Genomes
